# Home visits by family physicians during the end-of-life: Does patient income or residence play a role?

**DOI:** 10.1186/1472-684X-4-1

**Published:** 2005-01-27

**Authors:** Frederick I Burge, Beverley Lawson, Grace Johnston

**Affiliations:** 1 Department of Family Medicine, Dalhousie University, Halifax, NS, Canada; 2 School of Health Services Administration, Dalhousie University; and Cancer Care Nova Scotia, Halifax, NS, Canada

## Abstract

**Background:**

With a growing trend for those with advanced cancer to die at home, there is a corresponding increase in need for primary medical care in that setting. Yet those with lower incomes and in rural regions are often challenged to have their health care needs met. This study examined the association between patient income and residence and the receipt of Family Physician (FP) home visits during the end-of-life among patients with cancer.

**Methods:**

*Data Sources/Study Setting*. Secondary analysis of linked population-based data. Information pertaining to all patients who died due to lung, colorectal, breast or prostate cancer between 1992 and 1997 (N = 7,212) in the Canadian province of Nova Scotia (NS) was extracted from three administrative health databases and from Statistics Canada census records. *Study Design*. An ecological measure of income ('neighbourhood' median household income) was developed using census information. Multivariate logistic regression was then used to assess the association of income with the receipt of at least one home visit from a FP among all subjects and by region of residency during the end-of-life. Covariates in the initial multivariate model included patient demographics and alternative health services information such as total days spent as a hospital inpatient. *Data Extraction Methods*. Encrypted patient health card numbers were used to link all administrative health databases whereas the postal code was the link to Statistics Canada census information.

**Results:**

Over 45% of all subjects received at least one home visit (n = 3265). Compared to those from low income areas, the log odds of receiving at least one home visit was significantly greater among subjects who reside in middle to high income neighbourhoods (for the highest income quintile, adjusted odds ratio [OR] = 1.37, 95% confidence interval [CI] = 1.15, 1.64; for upper-middle income, adjusted OR = 1.19, 95%CI = 1.02, 1.39; for middle income, adjusted OR = 1.33, 95%CI = 1.15, 1.54). This association was found to be primarily associated with residency outside of the largest metropolitan region of the province.

**Conclusion:**

The likelihood of receiving a FP home visit during the end-of-life is associated with neighbourhood income particularly among patients living outside of a major metropolitan region.

## Background

In the last ten years, more and more of those dying of cancer in Canada are doing so out of hospital[1]. In Nova Scotia, a Canadian Maritime province with a total population of approximately 950,000 people, the proportion of cancer deaths occurring out of hospital has recently grown by fifty per cent[2]. This trend appears to be associated with a number of factors. More individuals with cancer are choosing to remain in the home setting, hospitals have down-sized thus reducing the number of beds available for end-of-life care [3-5], and there is growing availability of services in the community such as homecare and community based palliative care programs [6-8].

As this trend has developed, it has become even more important for patients and families to have access to medical care in the community. Such first line medical care, in Canada, is generally provided by a family physician, usually previously known to the patient. Initially those with terminal illness will obtain their medical care in the office or out patient setting. As they become sicker, however, there will come a time when getting from the home to the clinic office will be too difficult. At such a time, access to home visiting by a physician becomes very important [9-11]. Research has shown that access to a supportive family physician willing to make home visits is associated with a greater likelihood of a home death [12-14], as is access to a comprehensive palliative care program (PCP)[2].

There is evidence that those better off financially live longer and are in better health than poorer individuals[15,16]. Reasons for this have been postulated to include the fact that those with higher incomes have higher educational achievement, better living circumstances, and less risky health behaviours. Such better health may also be due, in part, to better access to services for those with fewer financial barriers. In Canada, such an association should not be the result of inadequacy of health services provided to those with lower incomes as our federal government has committed to the provision of a universal, accessible, comprehensive publicly administered health insurance system which aims to ensure that all residents have access to necessary hospital and physician services on a prepaid basis[17].

We wondered if access to terminal care home visiting by family physicians is better for those with higher incomes even in our publicly-funded health system. Therefore, the purpose of this study was to examine the association between income and the likelihood of receiving home visits by family physicians during the end-of-life among those with cancer. In addition, we examined the effect of regional residency, specifically residency in a major urban centre versus all other regions of NS (which are much smaller in size).

## Methods

### Data

Data for this retrospective, population-based study were obtained through the linkage of individual-level information extracted from four administrative health databases: (1) the Nova Scotia Cancer Centre Oncology Information System (OPIS) which includes the Nova Scotia Cancer Registry (NSCR) and provincial vital statistics information, (2) the Nova Scotia Medical Services Insurance Physician Services (MSIPS), (3) the Nova Scotia Hospital Admissions / Separations (HAS) file, and (4) the Queen Elizabeth II Health Sciences Center Palliative Care Program (PCP). The MSIPS provides a record of all services provided by physicians to residents of Nova Scotia whereas the HAS contains information relating to all hospital inpatient and outpatient stays and procedures. Because individual-level income information is not available from these sources, the Postal Code Conversion File (PCCF) and 1996 Statistics Canada census data were used to develop an 'ecologic-level' proxy for household income, enumeration area median (EAM) income quintiles or 'neighbourhood income'. These aggregate measures are derived from census information grouped by provincial enumeration areas or 'neighbourhoods'. The resulting quintiles are based on the median income value of each enumeration area. Evidence suggests the use of such proxies in population-based studies is a valid alternative in situations where household level information in not available[18]. It is, however, important to recognize that ecologic measures represent conceptually distinct measures of SES even when individual measures are available [19-21].

Encrypted patient health care numbers were used to link all four administrative health databases whereas the postal code was the link to the PCCF and Statistics Canada census information. Ethics approval for this project was provided by the Queen Elizabeth II Health Sciences Centre research ethics committee.

### Subjects

All adults identified on death certificates in the NSCR database as having died due to lung, colorectal, breast or prostate cancer death (International Classification of Diseases, 9^th ^revision [ICD9-CM]) from 1992 to 1997 were included as subjects. These four cancers represent the most common causes of cancer death in Nova Scotia.

### Measures

Patient characteristics included sex, date of birth, region of residency (Halifax regional municipality [HRM], all regions outside of HRM), date of initial cancer diagnosis, year of death (1992–1997) cancer cause of death (lung, colorectal, breast, prostate), and neighbourhood income categorized as provincial quintiles (lower, lower middle, middle, upper middle, upper). Almost 40% (39.5%) of Nova Scotia's population resides in the HRM which spans a primarily urban geographical region. Within HRM's boundaries are all of Nova Scotia's major tertiary health care centres, several community hospitals and many specialized care programs. Although regions outside of HRM also encompass many towns with regional and community health care facilities, they span a much larger, diverse, geographical area and may be considered to be relatively more rural than HRM.

Health services information was limited to each subject's 'survival time'. In our end-of-life research we have defined 'end-of-life' as the last 180 days of life, or if of shorter duration, from the date of initial cancer diagnosis to death. This six month time period is commonly used in end-of-life studies [22-24].

Total family physician home visits received during this survival time were counted and, due to the highly skewed distribution evidenced, also dichotomized to represent at least one home visit received or none. Additional health services of interest and potential covariates included the total number of ambulatory visits made to a family physicians by each subject, the total number of visits made by the subject to a specialist, the total number of days spent as a hospital inpatient, receipt of palliative radiotherapy, and whether or not the patient had been admitted to the PCP, a comprehensive palliative care program which has been operating since 1992. As an indirect measure of whether the patient was a resident of a long term care (LTC) facility during the end-of-life, a flag was created indicating whether a patient had received at least one family physician within a LTC centre.

### Analysis

Following descriptive statistics and the application of nonparametric tests to assess median differences and cross-tabulations with chi-square analyses for association, regression techniques were employed to estimate the effect of neighbourhood income (EAM income quintile) on the receipt of family physician home visits. Our initial regression analysis retained the total number of home visits as a continuous dependent variable and involved negative binomial regression where differences in survival time were accounted for as an offset variable. This was followed by logistic regression techniques where the probability of receiving at least one FP home visit versus no home visits was assessed. For both forms of regression, unadjusted analyses were followed by multivariate where the initial model included neighbourhood income in addition to sex, year of death, age, cancer cause of death, region of residency, the number of visits made to a medical specialist, the receipt of palliative radiotherapy and admission to the PCP as covariates. To account for the possibility that the subject may not have been 'at home' during their 'survival time' and hence unable to receive a home visit, our LTC residency flag and the total days spent as a hospital inpatient were added to the model.

To control for differences in 'survival time', the number of days from the initial cancer diagnosis date to death were categorized and added to the model. Subsequent modeling involved the sequential elimination of covariates and confounders found to no longer be significantly associated with the receipt of FP home visits in the multivariate model at the p = 0.05 level of significance.

Since our administrative data do not provide the ability to make adjustments for regional differences such as the availability of alternative health services, physician density or community resources, as an alternative, we stratified each analysis by region of residency.

All analyses were performed using SAS software[25].

## Results

In total, 7212 adults were identified from death certificate information as having died from lung, colorectal, prostate or breast cancer between 1992 and 1997 in Nova Scotia. Over 94% of these advanced cancer patients had seen a family physician at least once during the end-of-life. In total, home visits accounted for 29% of all ambulatory visits provided by family physicians with 3265 (45.3%) patients receiving at least one FP home visit. The total number of FP home visits received varied widely, from 0 to 89 with an average number of 2 (standard deviation [SD] 4.2) and median of 0. Table 1 records the number of home visits received within each neighbourhood income quintile and by region of residency. Although patients from upper and middle income neighbourhoods across all of Nova Scotia appear to receive a greater number of home visits than those from lower income neighbourhoods, examination by region of residency reveal that this gradient by income is primarily associated with residency outside of HRM. Furthermore, patients residing outside of HRM tend to receive fewer home visits in general (mean 1.75, SD 4.1; median 0, range 0–89) than those living within the metropolitan region (mean 2.53, SD 4.4; median 1, range 0–56). The differences between the mean and median number of visits by region of residency were significant at the p < 0.0001 level. Results were similar in the examination of home visits as a dichotomy. A greater proportion of patients residing in middle to upper income neighbourhoods received at least one home visit than those from lower income areas (Table 2). Again, after controlling for region of residency, this association was found only to apply to those residing in regions outside of HRM (p < 0.0001).

**Table 1:** Family physician home visits by neighbourhood income quintiles and region of residency

	**Family physician home visits Mean (standard deviation); Median (range)**		
		**Region of residency**	
		
**Neighbourhood income quintile**	**All adult Nova Scotians**	**Halifax regional municipality**	**All other regions**

Lower	1.67 (4.2); 0 (0–89)	2.30 (4.1); 0 (0–30)	1.53 (4.2); 0 (0–89)
Lower middle	1.77 (4.1); 0 (0–69)	2.60 (4.6); 1 (0–31)	1.60 (4.0); 0 (0–69)
Middle	2.25 (4.7); 0 (0–58)	2.96 (5.1); 1 (0–45)	2.02 (4.9); 0 (0–58)
Upper middle	2.15 (4.0); 0 (0–56)	2.44 (4.4); 1 (0–56)	1.95 (3.6); 0 (0–24)
Upper	2.42 (3.9); 1 (0–35)	2.36 (3.7); 1 (0–23)	2.65 (4.6); 0 (0–35)
All	2.00 (4.2); 0 (0–89)	2.53 (4.4); 1 (0–56)	1.75 (4.1); 0 (0–89)

Note: Mean and median visits differ significantly at the p < 0.0001 level

**Table 2:** Characteristics of Nova Scotians by receipt of home visits during the end-of-life, 1992–1997

**Characteristic**	Home visit receipt; No. (and %) of adult Nova Scotians*	
	
	**No home visit n = 3947**	**At least one home visit n = 3265**
**Neighbourhood income quintile^†^**		
Lower	1029 (60.0)	685 (40.0)
Lower middle	866 (58.4)	618 (41.6)
Middle	747 (51.5)	704 (48.5)
Upper middle	676 (52.3)	610 (47.4)
Upper	399 (45.2)	483 (54.8)

**Sex^†^**		
Male	2323 (56.9)	1763 (43.2)
Female	1624 (52.0)	1502 (48.1)

**Year of death^‡^**		
1992	643 (56.3)	499 (43.7)
1993	637 (54.8)	525 (45.2)
1994	655 (52.7)	588 (47.3)
1995	648 (52.1)	597 (48.0)
1996	679 (54.7)	562 (45.3)
1997	685 (58.1)	494 (41.9)

**Age group^§^**		
< 65 years	968 (55.6)	772 (44.4)
65–74 years	1165 (54.2)	986 (45.8)
75+ years	1814 (54.6)	1507 (45.4)

**Cancer case of death^†^**		
Lung	2094 (57.0)	1580 (43.0)
Colorectal	674 (55.1)	549 (44.9)
Breast	629 (50.6)	614 (49.4)
Prostate	550 (51.3)	522 (48.7)

**Survival time^†^**		
<61 days	935 (78.4)	258 (21.6)
61–120 days	307 (49.8)	310 (50.2)
121–180+ days	2705 (50.1)	2697 (49.9)

**Region of residency^†^**		
Halifax regional municipality	1082 (47.2)	1211 (52.8)
All other regions of Nova Scotia	2855 (58.2)	2049 (41.8)

**Visit within a long term care center (LTC)^†^**		
None	3421 (53.6)	2968 (46.5)
At least one LTC visit	526 (63.9)	297 (36.1)

**Specialty visits^‡^**		
0–2	1066 (57.5)	789 (42.5)
3–6	1025 (54.4)	859 (45.6)
7–13	736 (54.8)	608 (45.2)
14+	1120 (52.6)	1009 (47.4)

**Total days as hospital inpatient^‡^**		
0	568 (55.4)	458 (44.6)
1–12	1247 (55.3)	1007 (44.7)
13–31	1132 (52.0)	1044 (48.0)
32+	1000 (57.0)	756 (43.1)

**Admission to palliative care program^†^**		
No	3342 (59.2)	2301 (40.8)
Yes	605 (38.6)	964 (61.4)

**Received palliative radiation^†^**		
No	3085 (56.8)	2346 (43.2)
Yes	862 (48.4)	919 (51.6)

* Total number of patients by characteristic may vary due to missing values. Proportions are row percentages and may total more than 100 due to rounding.

^†^Characteristic is associated with receipt of home visit (p < 0.001)

^‡^Characteristic is associated with receipt of home visits (p < 0.05)

^§^No significant association demonstrated

Subject characteristics and health service utilization by receipt of at least one home visit are displayed in Table 2. In addition to patients who received at least one FP home visit tending to reside in higher income neighbourhoods, they also were more likely to be female, have a breast cancer cause of death, survived at least 61 days from their initial cancer diagnosis date, did not receive a FP visit within a LTC facility, made more than 14 specialty visits, spent 13–31 days as a hospital inpatient during their survival time, received palliative radiotherapy, and were admitted into the PCP.

Regression results incorporating the total number of home visits using negative binomial regression and those derived from logistic techniques assessing the log odds of receiving at least one home visit compared to none proved similar. Therefore, for ease of presentation, we present the logistic regression results only.

Displayed in Table 3 are the adjusted multivariate logistic regression results examining the effect of 'neighbourhood' income and additional predictors on the receipt of at least one FP home visit during the end-of-life among all advanced cancer patients and by region of residency. Examination of the crude odds ratios (OR) and related confidence intervals (CI) indicate the log odds of receiving at least one home visit was significantly greater among subjects who reside in middle to high income neighbourhoods compared to those from low income. Following adjustments for all other significant predictors retained in the model, this significant association remained, although less strongly. Compared to advanced cancer patients from lower income neighbourhoods, those from upper income neighbourhoods were 37% more likely to receive at least one FP home visit (adjusted odds ratio [OR] 1.37, 95% confidence interval [CI] 1.15, 1.64). Cancer patients from the upper-middle and middle income neighbourhoods were also significantly more likely to have received at least one family physician home visit than those from the lowest income area (for upper-middle income adjusted OR 1.19, 95%CI 1.02, 1.39; for middle income adjusted OR 1.33, 95%CI 1.15, 1.54). However, this association is not experienced equally across the province. Although patients residing in the large metropolitan region of HRM tend to receive more FP home visits in general, the receipt of such visits are not associated with neighbourhood income. In contrast, among patients living outside the HRM, those from upper income neighbourhoods were more than twice as likely to receive a FP home visit than others residing in lower neighbourhood income areas (adjusted OR 2.23; 95%CI 1.63, 3.07).

**Table 3:** Odds of receiving a home visit by income and other characteristics for Nova Scotia overall and by region

**Predictor**	**Nova Scotia overall**		**Halifax Regional Municipality [HRM]**	**All regions outside of HRM**
	
	**Crude OR**	Adjusted* OR (and 95% CI)	Adjusted* OR (and 95% CI)	Adjusted* OR (and 95% CI)
**Neighbourhood income quintile**				
Low	1.0	1.0 (-)	1.0 (-)	1.0 (-)
Lower middle	1.07	1.04 (0.90, 1.21)	1.42 (0.99, 2.03)	0.98 (0.84, 1.16)
Middle	1.42	1.33 (1.15, 1.54)	1.40 (1.00, 1.94)	1.30 (1.10, 1.53)
Upper middle	1.36	1.19 (1.02, 1.39)	1.21 (0.90, 1.64)	1.20 (0.99, 1.44)
Upper	1.82	1.37 (1.15, 1.64)	1.18 (0.88, 1.56)	2.23 (1.63, 3.07)

**Survival time**				
<61 days	1.0	1.0 (-)	1.0 (-)	1.0 (-)
61–120 days	3.66	3.83 (3.08, 4.77)	3.91 (2.62, 5.82)	3.69 (2.81, 4.84)
121–180+ days	3.61	4.04 (3.45, 4.72)	3.64 (2.76, 4.81)	4.21 (3.47, 5.11)

**Admission to palliative care program**				
No	1.0	1.0 (-)	1.0 (-)	1.0 (-)
Yes	2.30	2.25 (1.97, 2.56)	3.05 (2.49, 3.74)	1.47 (1.15, 1.86)

**Visit within a long term care center (LTC)**				
None	1.0	1.0 (-)	1.0 (-)	1.0 (-)
At least one LTC visit	0.65	0.55 (0.46, 0.65)	0.47 (0.34, 0.64)	0.60 (0.48, 0.75)

**Age group**				
< 65 years	1.0	1.0 (-)	1.0 (-)	1.0 (-)
65–74 years	1.06	1.25 (1.09, 1.44)	1.24 (0.97, 1.59)	1.28 (1.08, 1.51)
75+ years	1.04	1.41 (1.24, 1.61)	1.94 (1.52, 2.49)	1.29 (1.09, 1.51)

**Total days as a hospital inpatient**				
0	1.0	1.0 (-)	1.0 (-)	1.0 (-)
1–12	1.0	1.06 (0.90, 1.26)	1.02 (0.76, 1.37)	1.07 (0.87, 1.30)
13–31	1.14	1.07 (0.91, 1.27)	1.04 (0.77, 1.40)	1.08 (0.87, 1.32)
32+	0.94	0.80 (0.67, 0.95)	0.87 (0.64, 1.20)	0.78 (0.63, 0.97)

**Year of death**				
1992	1.0	1.0 (-)	1.0 (-)	1.0 (-)
1993	1.06	1.05 (0.88, 1.25)	1.07 (0.78, 1.48)	1.02 (0.82, 1.27)
1994	1.16	1.17 (0.98, 1.40)	1.13 (0.81, 1.57)	1.16 (0.94, 1.43)
1995	1.19	1.10 (0.92, 1.31)	0.88 (0.63, 1.22)	1.18 (0.95, 1.45)
1996	1.07	0.94 (0.79, 1.13)	0.76 (0.55, 1.05)	1.02 (0.83, 1.27)
1997	0.93	0.82 (0.69, 0.98)	0.80 (0.57, 1.11)	0.82 (0.66, 1.01)
**Sex**				
Male	1.0	1.0	1.0 (-)	1.0 (-)
Female	1.22	1.15 (1.04, 1.28)	1.24 (1.03, 1.50)	1.11 (0.98, 1.25)

Note: OR = odds ratio, CI = confidence interval

*Adjusted for all other listed predictors

Among all advanced cancer patients, additional factors predictive of receiving at least one FP home visit included a longer length of survival (for 121 to more than 180 days survival: adjusted OR 4.04; 95%CI 3.45, 4.72), admission to the QEII Palliative Care Program (PCP) (adjusted OR 2.25, 95%CI 1.97, 2.56), older age (for those 75 years and older: adjusted OR 1.41; 95%CI 1.24, 1.61) and being female (adjusted OR 1.15; 95%CI 1.04, 1.28). Patients who were in LTC at some point during their survival period (adjusted OR 0.55; 95%CI 0.46, 0.65) and those who spent 32 or more days in hospital compared to none (adjusted OR 0.80; 95%CI 0.67, 0.95) tended to be less likely to receive at least one FP home visit. Over time, the likelihood of receiving a home visit tended to decline. However, after accounting for all other predictors in the model, year of death was not a major factor.

Cancer cause of death, receipt of physician specialty visits and undergoing palliative radiation were not associated with home visit receipt in the final multivariate model.

## Discussion

The neighbourhood income of those dying of cancer is associated with the likelihood of receiving a home visit during the end-of-life by a family physician in Nova Scotia. The association found, however, appears to be modified by region of residence for those who died of cancer. It appears that income plays less of a role in predicting home visits by a family physician for those who live in the larger, urban centre of Halifax Regional Municipality. Given the finding that patients followed by the QEII Palliative Care Program are also more likely to receive family physician home visits, we speculate that the urban centre may provide a collaborative 'team-care' advantage to cancer patients. The publicly-funded PCP may act to equalize the opportunity to stay at home and facilitate family physician home involvement in ways rural locations may not be able to. In previous work, income was not associated with location of death but region of residency was[2]. We have also found that those who live in higher income areas tend to use the emergency department less[24].

In the United Kingdom, Aylin and colleagues[26] found, for the general population, those in social class 1 (highest income) received the fewest home visits. Their study also revealed a dose-response relationship in that as one moves to lower income class, the more likely one is to have received a home visit. Our study shows some gradient element, albeit in the opposite direction, but not as clearly. McNiece and Majeed found home visiting rates among patients with highest income to be half that of those with the lowest income[27]. Their study results and ours were adjusted for age and sex, such that the relationships between income, age and sex cannot be confounding the results. Aylin postulates that the reason for greater home visiting among those with lower income may be due to a number of factors including increased morbidity, poorer access to a car, and differing expectations of the services supplied by their general practitioners[26]. Such factors should also hold true for cancer patients. Nevertheless, our findings are opposite to those of Aylin.

We hypothesize that when it comes to routine home visits for brief, episodic illnesses, the home visiting trend may be as Aylin suggests. However, for those who are at home and looking to stay at home with advanced cancer, there are more substantial financial issues driving whether this is likely to happen or not. In Nova Scotia, as in many Canadian settings, there is access to home visiting nurses and some other health professionals through the publicly-funded health system. However, as disease progresses, the ability of a family to support death at home depends on many other factors. These include the presence of a family member who can stay at home, the ability of a family member to manage the medications and symptom assessment along with health professionals, the cost of drugs which are paid for in hospital but not in the home (unless the patient has private health insurance or is 65 or more years old), the cost of equipment in the home, and possibly the cost of additional nursing or personal care workers in the home (variably covered by the public system, and only sometimes covered by private insurance)[17]. All of these factors point to the fact that those with greater income would be more likely to succeed at staying at home[7]. In addition, in more rural settings there is less access to specialized services such as palliative care programs.

The home visits provided by family physicians may therefore be a direct response to the other capacities of patients and families to stay at home rather than being *the *critically enabling feature. Thus, the home visit is essential but not sufficient and without the rest of the support required, the patient will not be able to stay at home, thereby resulting in fewer home visits.

Another interpretation is that those with lower incomes may actually make different choices about how they wish to receive health care and where they would like to spend their last days. Depending on the study cited, up to 80% of those with advanced cancer wish to spend their last days at home[7,28-30]. Grande and colleagues reported that those who lived in higher socioeconomic areas were more likely to die at home than their counterparts[31]. Sims found that those with cancer from social class IV and V (semi-skilled and unskilled occupations) were under-represented in deaths that occurred in hospices and homes when compared to those in social class I and II (professional occupations and managerial/technical occupations)[32]. All of this may be supported by any one or a combination of factors such as less desire to remain at home, less capacity (financial or otherwise) to remain at home or bias in the delivery of health service by professionals. Our study is the first we know of to show that the number of home visits made by family physicians to those at the end-of-life is also less for these individuals.

Home visiting has long been an element of continuity of care across settings (office, hospital, home, nursing home) provided by family physicians. Some would argue that home visits may be influenced by the geography in which the physician operates daily. As a result, physician travel patterns to and from the office (when home visiting often occurs) may not take them through low income neighbourhoods, thus reducing the likelihood of a visit. The work of Aylin[26] and McNiece[27], however, does not support this.

New initiatives are underway in Canada which may provide more opportunities for the enhanced presence of a range of health professionals in the homes of the dying. In response to the Romanow Commission[33], the federal government of Canada has initiated agreement with the provinces for them to provide coverage for enhanced home-based end-of-life care. In the future, we may see more nurses or nurse-practitioners making home visits as part of the community-based care team along with family physicians[34]. In rural or remote areas where there is a scarcity of family physicians, nurses and nurse practitioners with advanced assessment skills may play an even greater role.

Our study has limitations. As we are using routinely collected data used for administrative and billing purposes there may be biases operating. The data reflect those family physicians who bill for the services they provide. It should also reflect the "shadow-billing" of those on alternate payment mechanisms (estimated to be less than five per cent of family physicians at the time of the study) but in reality, these physicians may have less incentive to capture these fee codes and so may under-report home visiting slightly.

The data file used in this study was originally created for an alternate project looking at health service utilization among patients who died due to lung, colorectal, breast or prostate cancer. We were therefore limited to examining home visits provided to these patients only and are not able to report whether the use of family physician home services among those who died due to all other cancer causes is similar or different.

We are unable to adjust for homecare utilization (data did not exist for the study years), family member caregiving status (no data available) or account for additional insurance coverage (above provincial) which may have covered additional costs associated with drugs, home nursing, home equipment, etc. Our attempt to account for service availability by region is crude. HRM is more homogeneous with respect to services than our combination all other regions outside of HRM; however, the effect evidenced may, therefore, be a conservative estimate.

## Conclusions

And so, in conclusion, it appears that even in death those with fewer financial resources may be less likely to achieve the same access to health services as those better off. What does this mean for our care of the dying? We must examine carefully which elements are the cause of this inequality. If there is less desire among those with more financial barriers, we need to examine the origins of these desires. Is it fear of caring for those at home? Is it an established culture of caring to move loved ones to hospital at the end-of-life? These issues need identification if we are to support such families. If there truly are financial barriers to such things as drugs, equipment and personnel then we must redefine health policy to make these more accessible to those with fewer financial resources. And finally, we should attempt to understand whether or not there is any bias operating on the part of health professionals. All of these need further research if we are to ensure patients receive the care where they wish to as they approach death.

## Competing interests

The author(s) declare that they have no competing interests.

## Authors' contributions

FB and BL participated in the conceptualization and design of the project, the analysis and interpretation of the data, first-drafted the majority of the article, and incorporated co-author comments into the final draft. GJ participated in the interpretation of the data and the drafting and revising of the manuscript. All authors gave approval to this final version.

## Pre-publication history

The pre-publication history for this paper can be accessed here:



## References

[B1] Wilson DM, Anderson JC, Fainsinger RL, al. (1998). Social and health care trends influencing palliative care and the location of death in Twentieth-Century Canada, Final NHRDP Report.

[B2] Burge F, Lawson B, Johnston G (2003). Trends in the place of death of cancer patients, 1992-1997. C M A J.

[B3] Tully P, Saint-Pierre E (1997). Downsizing Canada's hospitals, 1986/87 to 1994/95. Health Reports.

[B4] Health NSD (2000). Transitions in care: Nova Scotia Department of Health Facilities Review. http://www.gov.ns.ca/health/facilities/default.htm.

[B5] Wilson DM, Truman CD (2001). Does the availability of hospital beds affect utilization patterns? The case of end-of-life care. Health Serv Manage Res.

[B6] Nova Scotia Department of Health (1997). Home care Nova Scotia: Update.

[B7] Rural Palliative Home Care Staff and Consultants (2001). A rural palliative home care model: The development and evaluation of an integrated palliative care program in Nova Scotia and Prince Edward Island. A Federal Health Transition Fund Project Report.

[B8] Burge F, Johnston G, Lawson B, Dewar R, Cummings I (2002). Population based trends in referral of the elderly to a comprehensive palliative care program. Palliat Med.

[B9] Association CM (1994). Strengthening the foundation: The role of the physician in primary health care in Canada.

[B10] McWhinney IR (1994). Caring for patients with cancer: Family physicians' role. Can Fam Physician.

[B11] McWhinney IR, Stewart MA (1994). Home care of dying patients: Family physicians' experience with a palliative care support team. Can Fam Physician.

[B12] McWhinney IR, Bass MJ, Orr V (1995). Factors associated with location of death (home or hospital) of patients referred to a palliative care team. C M A J.

[B13] Cantwell P, Turco S, Brenneis C, Hanson J, Neumann CM, Bruera E (2000). Predictors of home death in palliative care cancer patients. J Palliat Care.

[B14] Brazil K, Bedard M, Willison K (2002). Factors associated with home death for individuals who receive home support services: a retrospective cohort study. BMC Palliat Care.

[B15] Lynch JW, Smith GD, Kaplan GA, House JS (2000). Income inequality and mortality: Importance to health of individual income, psychosocial environment, or material conditions. BMJ.

[B16] Adler NE, Ostrove JM (1999). Socioeconomic status and health: What we know and what we don't. Ann NY Acad Sci.

[B17] Canada H (2001). Canada Health Act: Annual Report 2000-2001.

[B18] Mustard CA, Derksen S, Berthelot JM, Wolfson M (1999). Assessing ecologic proxies for household income: A comparison of household and neighbourhood level income measures in the study of population health status. Health & Place.

[B19] Geronimus AT, Bound J, Neidert IJ (1996). On the validity of using census geocode characteristics to proxy individual socioeconomic statistics. J Am Stat Assoc.

[B20] Geronimus AT, Bound J (1998). Use of census-based aggregate variables to proxy for socioeconomic group: Evidence from national samples. Am J Epidemiol.

[B21] Kaplan GA (1996). People and places: Contrasting perspectives on the association between social class and health. Int J Health Serv.

[B22] Somogyi-Zalud E, Zhong Z, Hamel MB, Lynn J (2002). The use of life-sustaining treatments in hospitalized persons aged 80 and older. JAGS.

[B23] Latimer EA, Verrilli D, Welch WP (1999). Utilization of physician services at the end of life: Differences between the United States and Canada. Inquiry.

[B24] Burge F, Lawson B, Johnston G (2003). Family physician continuity of care and emergency department use in end-of-life cancer care. Med Care.

[B25] SAS Institute Inc (1999). SAS/STAT Version 8.2.

[B26] Aylin P, Majeed FA, Cook DG (1996). Home visiting by general practitioners in England and Wales. BMJ.

[B27] McNiece R, Majeed A (1999). Socioeconomic differences in general practice consultation rates in patients aged 65 and over: prospective cohort study. BMJ.

[B28] Dunlop RJ, Davies RJ, Hockley JM (1989). Preferred versus actual place of death: A hospital palliative care support team experience. Palliat Med.

[B29] Townsend J, Frank AO, Fermont D, Dyer S, Karran O, Walgrove A, Piper M (1990). Terminal cancer care and patients' preference for place of death: A prospective study. BMJ.

[B30] Higginson IJ, Sen-Gupta GJA (2000). Place of care in advanced cancer: A qualitative systematic literature review of patient preferences. J Palliat Med.

[B31] Grande GE, Addington-Hall JM, Todd CJ (1998). Place of death and access to home care services: Are certain patient groups at a disadvantage?. Soc Sci Med.

[B32] Sims A, Radford J, Doran K, Page H (1997). Social class variation in place of cancer death. Palliat Med.

[B33] Romanow RJ (2002). Building on values: The future of health care in Canada.

[B34] Canada H (2004). A 10-year plan to strengthen health care. http://www.hc-sc.gc.ca/english/hca2003/fmm/index.html.

